# Prevalence and trend of anaemia and haemoglobinopathy among pregnant indigenous women in Kuala Kangsar, Perak: A retrospective observational study

**DOI:** 10.51866/oa.1049

**Published:** 2026-05-25

**Authors:** Jazlan Jamaluddin, Thenmoli Palaniyappan, Sofiah Zainal Abidin, Gayathri Kathitasapathy, Mohamad Zikri Mohamad Isa, Paream Kaur, Husna Maizura Ahmad Mahir

**Affiliations:** 1 Department of Primary Care Medicine, Faculty of Medicine, Universiti Malaya, Kuala Lumpur, Malaysia.; 2 Social Accountability in Health Unit @ Universiti Malaya (SAHUM), Faculty of Medicine, Universiti Malaya, Kuala Lumpur, Malaysia.; 3 Klinik Kesihatan Kuala Kangsar, Jalan Sultan Idris Shah 1, Kuala Kangsar, Perak, Malaysia.; 4 Klinik Kesihatan Padang Rengas, Padang Rengas, Kuala Kangsar, Perak, Malaysia.; 5 Klinik Kesihatan Karai, Enggor, Karai, Kuala Kangsar, Perak, Malaysia.; 6 Klinik Kesihatan Lintang, Sungai Siput (U), Lintang, Kuala Kangsar, Perak, Malaysia.; 7 Klinik Kesihatan Simee, Jalan Kompleks Sukan, Ipoh, Perak, Malaysia.; 8 Perak State Health Department, Ministry of Health Malaysia, Jalan Koo Chong Kong, Ipoh, Perak, Malaysia.

**Keywords:** Anaemia, Haemoglobinopathies, Indigenous peoples, Malaysia, Haemoglobin E

## Abstract

**Introduction:**

Anaemia during pregnancy is a global health concern, particularly among indigenous populations such as the Orang Asli (OA) in Malaysia. Haemoglobinopathies further exacerbate this issue. This study aimed to examine the prevalence of anaemia and haemoglobinopathy and its associated factors among pregnant OA women.

**Methods:**

A retrospective observational study was conducted among pregnant OA women in Kuala Kangsar, Malaysia. Medical records from 2018 to 2022 were reviewed using a standardised data collection form. Women who were lost to follow-up, had transferred care or had pregnancies ending at or before 24 weeks of gestation were excluded. Descriptive analysis, trend analysis and multivariable logistic regression were performed.

**Results:**

A total of 766 women were included. The prevalence of anaemia during pregnancy was 44.6%, with 9.4% of the cases occurring before delivery. Haemoglobinopathy was identified in 22.7% of the cohort, with a significant increase observed over 5 years. The most common haemoglobinopathy was heterozygous haemoglobin E (HbE) (14.2%), followed by homozygous HbE (6.5%). HbE homozygous status and parenteral iron dextran use were significantly associated with anaemia before delivery.

**Conclusion:**

Anaemia and haemoglobinopathy are highly prevalent among pregnant OA women. Identifying their associated factors is crucial for the development of early screening programmes and appropriate management strategies to improve maternal and foetal health outcomes.

## Introduction

Anaemia is a global public health concern, affecting both developed and developing countries. It is the most common nutritional deficiency among pregnant women, posing substantial risks of maternal and foetal morbidity and mortality, including preterm birth, low birth weight and perinatal mortality. Iron-deficiency anaemia (IDA) remains the most common type of anaemia during pregnancy worldwide, accounting for the majority of cases, particularly in low- and middle- income countries. A Malaysian primary care study reported that IDA accounted for approximately 51% of anaemia cases among antenatal mothers.^1^ Anaemia in pregnancy can also impair the baby’s growth and development due to decreased iron stores. According to the World Health Organization (WHO), anaemia affects approximately 1.62 billion people worldwide, with the highest prevalence observed among pregnant women and young children. While the global prevalence of anaemia in women of reproductive age has remained stagnant since 2000, the prevalence among pregnant women decreased slightly, with 36.5% of pregnant women globally affected by anaemia in 2019, compared with 40.05% in 2016.^2,3^ Southeast Asia has one of the highest prevalence rates, with nearly half of its pregnant women affected by anaemia, accounting for a third of all pregnant women with anaemia worldwide. Despite global efforts, such as the 65 th World Health Assembly’s target to reduce anaemia prevalence among women of reproductive age by 50% by 2025, current progress remains insufficient.^4,5^

Haemoglobinopathy, a genetic disorder affecting the structure or production of haemoglobin, is another contributing factor to anaemia in pregnancy. Common haemoglobinopathies, such as thalassaemia and sickle cell disease, are prevalent in malaria-endemic regions such as Southeast Asia. In Malaysia, the estimated prevalence of haemoglobinopathy according to its subtypes is 4.5% for P-thalassaemia, 4.9% for a-thalassaemia and 5.5% for haemoglobin E (HbE) trait.^5^ In communities where consanguineous marriage is relatively common, such as the indigenous Orang Asli (OA) in Malaysia, a substantial burden of haemoglobin variants has been reported.^6^ In one screening study of 378 OA individuals, 256 haemoglobinopathy cases were identified, with heterozygous HbE carriers being the most common subtype.^7^ Offspring from such marriages may inherit two abnormal genes, resulting in severe forms of the disease, including chronic anaemia and organ damage.^8,9^

The OA, or ‘original peoples’ in Malay, is a collective term used to refer to the indigenous people of Peninsular Malaysia. They are divided into three main groups based on their language and cultural differences: the Negrito, Senoi and Proto-Malay, each of which has various sub-ethnic groups.^7^ Despite Malaysia’s socio-economic development, the OA population continues to face substantial health challenges, including high rates of anaemia and haemoglobinopathy. Among pregnant OA women, the prevalence of anaemia has been reported at 64%, while rates for other OA populations range from 33.0% to 61.1%.^10,11^ Limited healthcare access, geographical isolation, socio-economic disparities and cultural barriers exacerbate these health issues. However, recent data on anaemia and haemoglobinopathy prevalence and trends among pregnant OA women in Malaysia are lacking, necessitating updated research to inform healthcare interventions. According to a previous systematic review, there have been no studies on the impact of genetic factors, such as haemoglobinopathy and anaemia. This represents a substantial gap in the current literature, as recessive genes linked to inherited diseases are likely to emerge in these populations over time.^11^ Therefore, this study aimed to examine the prevalence of anaemia and haemoglobinopathy and its associated factors among pregnant OA women. The findings will assist healthcare teams, especially in primary care, in appropriately managing anaemia among pregnant OA women. Understanding the factors associated with the prevalence of anaemia among pregnant OA women is crucial for the development of effective interventions to reduce the burden of these disorders.

## Methods

### Study design and setting

This retrospective observational study was performed among pregnant OA women in Kuala Kangsar, which is one of the nine districts in Perak State, Malaysia, with one of the largest OA populations, predominantly from the Senoi ethnic group, accounting for 16.6% of the state’s total OA population.^12^ In Malaysia, health services for the OA community are mainly managed by the Ministry of Health’s Orang Asli Mobile Team (or Pasukan Bergerak Orang Asli [PBOA]) using land and air transport to locations that have been identified in rural OA Settlement Post and Regrouping Plan areas. In Kuala Kangsar, the mobile team was established since 1998 to include comprehensive healthcare services such as maternal and child health including antenatal care, family planning, infant care and childcare; outpatient services including both non-communicable and communicable diseases; dental services; and home visits. Currently, the PBOA team in Kuala Kangsar consists of two teams, Team A and Team B. Each team consists of one doctor, one medical assistant, one pharmacist or assistant pharmacy officer, three nurses and two drivers. Two four-wheel drive vehicles for each team are used to bring the team with medicine supplies, vaccines, treatment cards and tools to be used during clinic sessions. The coverage of the PBOA team in Kuala Kangsar involves visits to the OA settlements of Piah, Yum, Legap, Perwor, Poi and Kuala Mu.

During visits of the PBOA team, pregnant OA women go to the pre-specified post to receive their antenatal care. These women undergo assessment, relevant point-of-care investigations including measurement of their haemoglobin level and management by the PBOA team. They usually continue their follow-up until delivery, where they are offered to be placed in a transit complex for post-natal care. These visits are documented manually using standardised pregnancy health record books by the Ministry of Health in all clinics. The records are kept in the main office of the PBOA team in Kuala Kangsar and are brought separately for visits to each OA settlement. There are also other pregnant OA women living in other parts of Kuala Kangsar. These OA settlements are also referred to as ‘fringes’, defined as communities located on the outskirts of towns or in semi-urban areas where residents live in close proximity to the mainstream population but outside traditional rural villages or gazetted OA settlements. These women are usually followed up at one of the eight health clinics in the district according to their address. The pregnancy health record books for these women are kept in the record room of the respective health clinics. For this study, all pregnant OA women who were under follow-up in primary care health clinics in the district from 1 January 2018 to 31 December 2022 were included. Those who were lost to follow-up, those whose care was transferred to other clinics and those whose pregnancy ended at or less than 24 weeks of gestation (miscarriage) were excluded. Medical records of all pregnant OA women were reviewed, and data were collected using a standardised data collection form. In accordance with the WHO guidelines, anaemia before delivery, defined as haemoglobin <11.0 g/dL at the last documented antenatal after 35 weeks or term haemoglobin measurement.^4^ The primary outcome was the presence of anaemia before delivery.

The sample size was calculated using OpenEpi, version 3.01, update 47 on 06/04/2013, for ‘Sample Size for Frequency in a Population’, available at https://www.openepi.com/SampleSize/SSPropor.htm. Approximately 12,281 OA individuals live in Kuala Kangsar, and 18.3% of OA women have anaemia.^12,13^ Therefore, 226 patients were required to be studied to estimate the prevalence of anaemia among pregnant OA women with an absolute precision of ±5% and a two-sided confidence interval (CI) of 95%. However, we included all pregnant OA women under health clinic follow-up in Kuala Kangsar, Perak.

### Data analysis

Data were analysed using IBM SPSS Statistics, version 27.0 for Windows (IBM, Somers, NY, USA). Data of pregnant OA women who visited the selected PBOA antenatal clinics were deidentified and analysed as a cohort. Missing data were treated with listwise deletion in subsequent analyses. Categorical data were presented as numbers and percentages, while numerical data were described as medians and interquartile ranges (IQRs), as all numerical data were non-parametric based on the Shapiro–Wilk test results. The Cochran–Mantel–Haenszel test for trend was used to examine the temporal trends of anaemia and haemoglobinopathy from 2018 to 2022. Between- group differences were evaluated using the chi-square test or Fisher’s exact test, as appropriate. Multivariable logistic regression was performed using the entry method to investigate the factors associated with anaemia at term. These analyses were adjusted for baseline demographic and clinical characteristics measured prior to the outcomes. Variables with P-values of <0.20 were included in the final analysis. Collinearity between variables was ruled out before covariates were introduced into the model. Goodness of fit was tested using the Hosmer–Lemeshow test, and odds ratios with 95% CIs were computed. All reported P-values were two-sided, and a P-value of <0.05 was considered statistically significant.

## Results

During the study period, 796 records were identified. After women whose pregnancies ended at ≤24 weeks of gestation (n=30) were excluded, a total of 766 women were included in the analysis. The median age of the women was 25.0 years (IQR=9.0), and most of them were married (86.6%), with fewer than five pregnancies (88.3%). The median body mass index was 27.0 kg/m^2^ (IQR=7.7), and the majority were overweight (31.6%) and obese (47.1%). One- fifth of the women had diabetes. The most common haemoglobinopathy identified was HbE heterozygous trait (14.2%), while 6.5% of the cases were classified as homozygous HbE. Among the women with haemoglobinopathies, concomitant IDA was identified in 16 cases, including nine with HbE heterozygous trait, five with homozygous HbE and two with other haemoglobin variants. Most women were prescribed with Zincofer® (85.0%) as their iron tablets, while only 50 women (6.5%) were administered with parenteral iron dextran (Table 1).

**Table 1:** Characteristics of the pregnant OA women included according to the presence of anaemia before delivery.

Characteristic	n (%)	Anaemia before delivery		Crude OR (95% CI)	P-value
		Yes	No		
**Residence** Rural Fringe Others	397 (51.8) 364 (47.5) 5 (0.7)	33 (50.8) 31 (47.7) 1 (1.5)	301 (48.1) 322 (51.4) 3 (0.5)	Reference 0.88 (0.53-1.47) 3.04 (0.31-30.07)	0.621 0.342
**Age, year** <20 20-34 ≥35	125 (16.3) 550 (71.8) 91 (11.6)	10 (15.4) 49 (75.4) 6 (9.2)	91 (14.5) 461 (73.6) 74 (11.8)	1.03 (0.51-2.12) Reference 0.76 (0.31-1.84)	0.927 0.548
**Marital status** Married Single	663 (86.6) 100 (13.1)	57 (87.7) 8 (12.3)	549 (88.0) 75 (12.0)	Reference 1.03 (0.47-2.24)	0.946
**Gravida** <5 ≥5	676 (88.3) 90 (11.7)	61 (93.8) 4 (6.2)	550 (87.9) 76 (12.1)	Reference 0.48 (0.17-1.34)	0.160
**Body mass index at booking, kg/m^2^** Underweight (<18.5) Normal (18.5-22.9) Overweight (23.0-27.4) Obese (>27.5)	12 (1.6) 151 (19.7) 242 (31.6) 361 (47.1)	0 (0) 15 (23.1) 17 (26.2) 33 (50.8)	9 (1.4) 115 (18.4) 202 (32.3) 300 (47.9)	NA Reference 0.65 (0.31-1.34) 0.84 (0.44-1.61)	NA 0.240 0.606
**Comorbidity** Diabetes Hypertensive disease	160 (20.9) 47 (6.1)	18 (27.7) 5 (7.7)	128 (20.4) 37 (5.9)	1.49 (0.84-2.65) 1.33 (0.50-3.50)	0.175 0.568
**Medication** Aspirin Calcium carbonate Metformin Insulin Methyldopa Labetalol	39 (5.1) 37 (4.8) 39 (5.1) 20 (2.6) 17 (2.2) 6 (0.8)	4 (6.2) 3 (4.6) 8 (12.3) 3 (2.7) 2 (3.1) 2 (3.1)	32 (5.1) 32 (5.1) 29 (4.7) 17 (4.6) 10 (1.6) 4 (0.6)	1.22 (0.42-3.56) 0.90 (0.27-3.02) 2.87 (1.25-6.56) 1.73 (0.49-6.08) 1.96 (0.42-912) 4.94 (0.89-27.48)	0.767 0.862 0.009 0.390 0.393 0.068
**Aetiology of anaemia** Iron deficiency Dilutional anaemia Unknown	95 (12.4) 24 (3.1) 103 (13.4)	17 (26.6) 1 (1.6) 11 (17.2)	66 (27.6) 20 (8.4) 74 (31.0)	0.95 (0.51-1.77) 0.17 (0.02-1.32) 0.46 (0.23-0.94)	0.867 0.091 0.032
**Haemoglobinopathy status** Not diagnosed HbE heterozygous trait Homozygous HbE Others	592 (77.3) 109 (14.2) 50 (6.5) 15 (2.0)	24 (36.9) 10 (15.4) 26 (40.0) 5 (7.7)	503 (80.4) 90 (14.4) 24 (3.8) 5 (1.4)	Reference 2.33 (1.08-5.04) 22.71 (11.39-45.25) 11.64 (3.62-37.42)	0.032 <0.001 <0.001
**Haematinic** Zincofer® Ferrous fumarate Iberet® Obimin^®^ Maltofer®	651 (85.0) 442 (57.7) 162 (21.1) 131 (17.1) 49 (6.4)	52 (80.0) 38 (58.5) 30 (46.2) 8 (12.3) 16 (24.6)	537 (85.8) 349 (55.8) 119 (19.0) 113 (18.1) 30 (4.8)	0.66 (0.35-1.27) 1.12 (0.67-1.88) 3.65 (2.16-6.19) 0.64 (0.30-1.37) 6.49 (3.31-12.72)	0.214 0.675 <0.001 0.250 <0.001
**Parenteral iron dextran**	50 (6.5)	19 (29.7)	30 (12.6)	2.94 (1.52-5.68)	<0.001
**Haemoglobin level before delivery, g/dL** ≥11.0 <11.0	626 (90.6) 65 (9.4)				
**Severity of anaemia** Mild Moderate	42 (64.6) 23 (35.4)				

CI: confidence interval, HbE: haemoglobin E, NA: not applicable, OR: odds ratio

**Figure 1:**
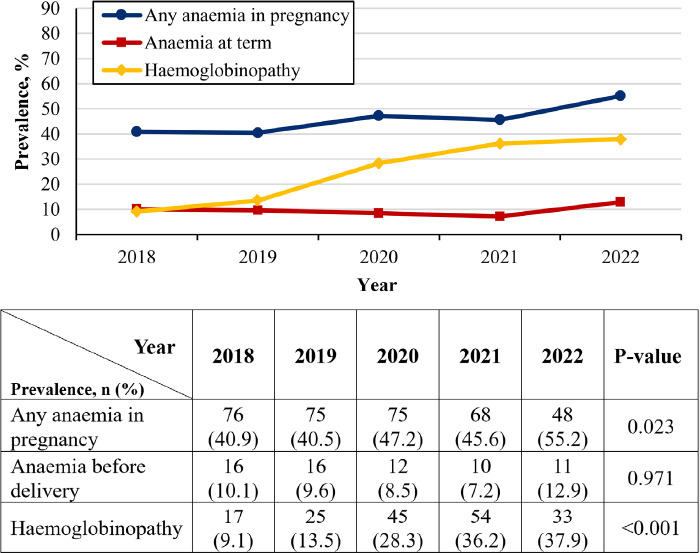
Proportion of anaemia and haemoglobinopathy among the pregnant OA women over the past 5 years.

The overall prevalence ofanaemia during pregnancy was 44.6% (95% CI=41.1–48.2). Approximately 9.4% of the women (95% CI=7.3–11.8) had anaemia at term. Haemoglobinopathy was detected in 22.7% (95% CI=19.8–25.8) of pregnant OA women, with a significant increase observed over the past 5 years. The prevalence of anaemia during pregnancy significantly increased in the past 5 years, especially after 2019; however, the prevalence of anaemia at term remained stable (Figure 1).

In the subsequent analysis, only 691 pregnant women with documented haemoglobin levels before delivery were included, as haemoglobin measurements at delivery were unavailable for the remaining cases. The median haemoglobin level at term was 12.0 g/dL (IQR=1.3). In the univariate analysis, 10 variables were found to have a P-value of <0.20: gravida, diabetes, aetiology of anaemia (dilutional anaemia and unknown aetiology), haemoglobinopathy status and prescription of metformin, labetalol, Iberet®, Maltofer® and parenteral iron dextran (Table 1). The multivariable analysis showed that only HbE homozygous status (adjusted odds ratio [aOR]=7.38, 95% CI=2.74– 19.89, P<0.001) and parenteral iron dextran use (aOR=3.19, 95% CI=1.30–7.80, P=0.011) were associated with anaemia before delivery among the pregnant OA women (Table 2).

**Table 2:** Factors associated with anaemia before delivery among the pregnant OA women.

Variable	Beta	Wald statistics (df)	aOR (95% CI)	P-value
**Gravida** <5 >5	-1.063	2.264 (1)	Reference 0.35 (0.09-1.38)	0.132
**Diabetes** No Yes	0.355	0.547 (1)	Reference 1.43 (0.56-3.66)	0.460
**Metformin use** No Yes	0.395	0.283 (1)	Reference 1.49 (0.35-6.38)	0.595
**Labetalol use** No Yes	2.780	3.761 (1)	Reference 16.11 (0.97-267.35)	0.052
**Dilutional anaemia** No Yes	-1.456	1.746 (1)	Reference 0.23 (0.03-2.02)	0.186
**Unknown aetiology** No Yes	0.167	0.118 (1)	Reference 1.18 (0.46-3.07)	0.731
**Haemoglobinopathy status** Not diagnosed HbE heterozygous trait Homozygous HbE Others	-0.088 1.998 1.942	0.030 (1) 15.980 (1) 7.212 (1)	Reference 0.92 (0.34-2.47) 7.38 (2.74-19.89) 6.97 (1.69-28.76)	0.863 <0.001* 0.007*
Iberet® use No Yes	-0.461	1.295 (1)	Reference 0.63 (0.28-1.40)	0.255
Maltofer® use No Yes	0.389	0.637 (1)	Reference 1.48 (0.56-3.84)	0.425
Parenteral iron dextran use No Yes	-1.991	6.428 (1)	Reference 3.19 (1.30-7.80)	0.011*

The model reasonably fit well (Hosmer-Lemeshow test: chi-square=2.867; P=0.942); model assumptions were met; there were no significant interactions and multicollinearity problem; the model explained 17.5% (Cox and Snell R^2^) to 27.2% (Nagelkerke R^2^) of the variance for the presence of anaemia before delivery. The model correctly classified the percentage of cases with a sensitivity of 83.8% and a specificity of 66.7%. *Statistical significance at P<0.05. aOR: adjusted odds ratio, CI: confidence interval.

## Discussion

The prevalence of anaemia during pregnancy among the OA women in our study (44.6%) is slightly higher than the global prevalence but falls within the range of anaemia prevalence among pregnant women in Malaysia. It is also higher than that reported among OA women in a previous Malaysian community-based study.^3,13-15^ This highlights the increased risk of anaemia in pregnant women, particularly among marginalised communities such as the OA population. Conversely, the prevalence of anaemia at term is lower in our study than in other studies, possibly due to effective interventions for correcting anaemia in this population.^14^ However, the prevalence of haemoglobinopathy among the pregnant OA women in our study (22.7%) is notably high compared with the national prevalence.^5,8,15,16^ This emphasises the need for targeted interventions to address this genetic disorder in this population. While our analysis showed a significant increasing trend in the diagnosis of haemoglobinopathy over the past 5 years, especially after 2019, there had been a minimal increase in the trend of anaemia at term during the same period. This increase may be attributed to improved awareness and the presence of local guidelines for managing anaemia and haemoglobinopathy, which may have facilitated earlier detection of anaemia, diagnosis of haemoglobinopathy and treatment of nutritional anaemia where relevant.

Our study found a significant association of anaemia during pregnancy with HbE homozygous status but no association with HbE heterozygous status. This finding aligns with previous reports indicating an increased risk of anaemia with certain haemoglobinopathy subtypes, such as homozygous HbE.^17^ P-thalassaemia mutations are relatively population-specific, wherein each ethnic group displays its own set of common mutants. HbE occurs from a mutation at Position 26 of the P-globin chain (Glu --> Lys).^18^ It is most commonly found in Southeast Asia and displays a wide spectrum of severity depending on different interactions of P-thalassaemia alleles of varying severity, co-inheritance of a-thalassaemia alleles and the relative level of persistent foetal haemoglobin synthesis.^17^ HbE is known to be unstable in vitro, potentially contributing to the severity of anaemia.^18^ However, the lack of association with HbE heterozygous status in this study requires further investigation. Anaemia associated with haemoglobinopathies is generally not expected to respond to iron supplementation. However, in our cohort, a subset of the participants with haemoglobinopathies also had concomitant IDA. The presence of concurrent iron deficiency may partly explain the improvement in haemoglobin levels observed following iron supplementation in this subgroup. A previous study showed no difference in iron utilisation and absorption between adults with HbE heterozygotes and controls, whereas significant differences were observed in individuals with a- and P-thalassaemia.^19^ However, there is still a scarcity of studies, particularly regarding HbE trait, to suggest differing mechanisms underlying anaemia in individuals with various haemoglobinopathy subtypes. A study in children showed that HbE homozygotes with IDA responded well to 2 months of iron supplementation, resulting in a significant increase in serum ferritin levels and most haematological parameters, except for red cell distribution width and red blood cell count.^20^ The average haemoglobin level achieved after iron supplementation was 11.4 g/dL. Both thalassaemia with iron overload and IDA co-exist in the same population in Southeast Asia, highlighting the importance of testing for and treating IDA among patients with HbE diseases with the goal of achieving a haemoglobin level over 11.0 g/ dL.^17^ Another possible explanation for persistent anaemia despite parenteral iron therapy is the high prevalence of HbE homozygous status in this population. Individuals with HbE-related disorders may have anaemia that is not primarily driven by IDA, which could result in a suboptimal response to iron therapy.

Nevertheless, our analysis found that the OA women who were administered with parenteral iron had 3.19 times higher odds of having anaemia before delivery. This association may be attributed to the high prevalence of haemoglobinopathy in this population, as individuals with certain haemoglobinopathy subtypes such as a- and P-thalassaemia heterozygous groups may not respond adequately to parenteral iron supplementation.^17^ It is important to perform iron studies before initiating parenteral iron therapy in pregnant OA women to ensure appropriate management of anaemia. This association also likely reflects confounding by indication. Women with more severe or persistent anaemia would have been more likely to receive parenteral iron therapy; therefore, this association should not be interpreted as suggesting that parenteral iron dextran increases the risk of anaemia. Since our study used a cross-sectional design, which precludes the establishment of causation, further research is needed to elucidate the underlying mechanisms driving this association. In contrast to parenteral iron, no other medications were found to be associated with anaemia during pregnancy among the OA women, including metformin. This finding is reassuring, particularly in light of previous concerns regarding metformin’s potential association with vitamin B12 deficiency.^21^ The absence of evidence of such an association in our study may be attributed to the relatively short-term use of metformin among the pregnant OA women. Similarly, our analysis did not find any other aetiology to be associated with anaemia at term, particularly IDA. This finding may be attributed to the effective treatment of IDA with haematinics and the prompt response to treatment observed in this population. However, further research is warranted to explore other potential contributors to anaemia at term among pregnant OA women.

Our study is one of the first to report the prevalence of haemoglobinopathy among pregnant OA women and to identify HbE homozygous status as significantly associated with anaemia during pregnancy. However, there are several limitations that should be acknowledged, including the crosssectional design, which prevents establishing causation, and the retrospective record analysis, which may have led to missing data and potential bias. Due to limitations in the retrospective records, it was not always possible to distinguish between previously known cases of haemoglobinopathy and cases newly detected during antenatal screening. Furthermore, our study focused on Kuala Kangsar and included only women who sought follow-up care there, limiting the generalisability of our findings to the broader OA population. The exclusion of women lost to follow-up or whose pregnancies ended early may introduce selection bias, also potentially limiting the generalisability of the findings.

Our findings highlight the need for targeted screening programmes to identify and manage anaemia and haemoglobinopathy among pregnant OA women, considering the high prevalence observed in this population. In Malaysia, the Ministry of Health conducts a thalassaemia screening programme targeting secondary school students, particularly those in Form 4 at approximately 16 years of age, as part of a national prevention strategy.^22,23^ However, this programme may not reach all students, particularly those in remote indigenous communities. Some OA students may not attend schools that participate in the programme or may discontinue schooling before reaching Form 4, limiting the effectiveness of this screening strategy. The high haemoglobinopathy prevalence suggests the need for expanded genetic counselling, targeted screening and community-based health education initiatives. Further evolution of discussions about the possibility of prenatal diagnosis as an interim method for the control of thalassaemia should also take place. Additionally, future research should investigate pregnancy outcomes among pregnant OA women with haemoglobinopathy compared with those with anaemia during pregnancy in the general population. Depending on the data, population- specific haemoglobin levels can be targeted accordingly with optimum maternal and foetal outcomes in mind. The development of more adequate approaches for the treatment of haemoglobinopathy such as gene-modifying agents must be explored. Moreover, efforts to improve access to education and healthcare services among OA communities are crucial in addressing the social determinants underlying the burden of anaemia and haemoglobinopathy in this population.

## Conclusion

Anaemia is common among pregnant OA women. However, the prevalence of haemoglobinopathy is higher than the national and global prevalence. The identification of associated factors, such as HbE homozygous status and parenteral iron dextran use, can be utilised to guide focused actions. This serves as a call to action for policymakers, healthcare providers and researchers to address the high rates of anaemia and haemoglobinopathy among pregnant women through targeted interventions and further research. Effective interventions for early detection and management of anaemia and haemoglobinopathy in this vulnerable group are needed to promote maternal and foetal health.

## Data sharing statement

Data will be made available upon reasonable request.

**How does this paper make a difference in general practice?**

- This study supports the need for targeted antenatal screening programmes for anaemia and haemoglobinopathy among pregnant Orang Asli (OA) women due to their high disease burden.
- It highlights gaps in current school-based thalassaemia screening, especially in rural and underserved OA communities.
- It highlights the need for future research to examine whether population-specific haemoglobin patterns are associated with maternal and neonatal outcomes.
- It informs improved access to culturally appropriate healthcare and nutritional interventions addressing social determinants.
- It guides future research comparing pregnancy outcomes between OA women with haemoglobinopathy and anaemia among the general population.
